# Cytokines levels, Severity of acute mucositis and the need of PEG tube installation during chemo-radiation for head and neck cancer - a prospective pilot study

**DOI:** 10.1186/1748-717X-5-16

**Published:** 2010-02-25

**Authors:** Amichay Meirovitz, Michal Kuten, Salem Billan, Roxolyana Abdah-Bortnyak, Anat Sharon, Tamar Peretz, Mordechai Sela, Moshe Schaffer, Vivian Barak

**Affiliations:** 1 Department of Oncology, Hadassah-Hebrew University Medical Center, Jerusalem, Israel; 2 School of Dental Medicine, Hadassah-Hebrew University, Jerusalem, Israel; 3 Division of Oncology, Rambam Health Care Campus and Faculty of Medicine, Technion-Israel Institute of Technology, Haifa, Israel; 4 Department of Maxillofacial Prosthetics, Hadassah-Hebrew University Medical Center, Jerusalem, Israel

## Abstract

**Background:**

The purpose of this pilot study was to detect a correlation between serum cytokine levels and severity of mucositis, necessitating installation of a percutaneous endoscopic gastrostomy tube (PEG) in head and neck (H&N) cancer patients receiving combined chemo-radiation therapy.

**Patients and Methods:**

Fifteen patients with H&N epithelial cancer were recruited to this study. All patients received radiotherapy to the H&N region, with doses ranging from 50-70 Gy. Chemotherapy with cisplatin, carboplatin, 5-fluorouracil and taxanes was given to high-risk patients, using standard chemotherapy protocols. Patients were evaluated for mucositis according to WHO common toxicity criteria, and blood samples were drawn for inflammatory (IL-1, IL-6, IL-8, TNF-α) and anti-inflammatory (IL-10) cytokine levels before and during treatment.

**Results:**

A positive correlation was found between IL-6 serum levels and severity of mucositis and dysphagia; specifically, high IL-6 levels at week 2 were correlated with a need for PEG tube installation. A seemingly contradictory correlation was found between low IL-8 serum levels and a need for a PEG tube.

**Conclusion:**

These preliminary results, indicating a correlation between IL-6 and IL-8 serum levels and severity of mucositis and a need for a PEG tube installation, justify a large scale study.

## Introduction

More than 60% of all squamous cell tumors of the head and neck are treated by ionizing irradiation or radio-chemotherapy and, more recently, by radiation combined with biological treatment (targeted therapy) such as Erbitux [1]. The most frequent side effect of treatment is mucositis that can appear in severe modes, especially in patients treated with radio-chemotherapy. This side effect can hinder the process of the treatment and, in the long-term, be followed by xerostomia [2]. Severe form of mucositis can occur anytime between week 2-6 of treatment, related to the chemotherapy regimen and the technique and fractionation of radiotherapy. The symptoms usually become more severe with treatment and remain for weeks after the end of therapy [1]. Severe mucositis is followed by other symptoms, such as pain, dysphagia, weight loss and other symptoms that can influence therapy [2]. To avoid cachexia and to provide necessary alimentation, installation of percutaneous endoscopic gastrostomy tubes (PEG) is performed [3]. This can be an expensive undertaking, as the cost in the USA is estimated at $3000 ± 1000 per patient.

The correlation between ending therapy because of side effects and therapy success has been shown by Alden et al [3]. The early detection of high-risk patients who might have severe mucositis and dysphagia can be very important from therapeutic and economic points of view [4]. The severity of the mucositis depends on various factors, such as total dose and dose per fraction, fractionation schedule (standard or altered), irradiation fields, type of chemotherapy, and individual mouth hygiene during and before treatment. Smoking and alcohol consumption during therapy will enhance severity. The variability of factors creates difficulty in determining which patients will develop severe mucositis. To avoid the consequences of side effects, most centers provide the installation of gastrostomy tubes to all head and neck (H&N) cancer patients. This installation is not always necessary but, to date, there is no method to determine in advance which patient will suffer from severe mucositis and dysphagia and which will not [5].

Mucositis has four phases [2]: a) inflammation; b) epithelial; c) ulcera; d) repair. The inflammation phase, also called the initial tissue injury phase, ends with the production of cytokines such as IL-1 and TNF-α [2]. The pro-inflammation cytokines IL-1 and TNF-α are present in high levels in blood and serum during inflammation, and anti-inflammation cytokines are at low levels. This cytokine balance is very important during the inflammation process [6]. A correlation between IL-1 and TNF-α levels and the response of the tumor to treatment was found in patients with breast cancer [7], with a better clinical response following low levels of IL-1 and TNF-α.

Markers, such as cytokeratin TPS, were found to be indicators of the response of the tumor to treatment and prognosis [7].

In squamous cell tumors of the H&N, Chen et al. found inflammatory cytokines that are pro-angiogenetic and immuno-regulators [8]. These cytokines are produced from the tumor cells themselves and can be an indication of the pathogenesis of the tumor [8]. In this regard, a correlation was found between high levels of inflammation cytokines IL-6 and IL-8 in the serum of patients suffering from squamous cell cancer of the H&N as a reaction to stimulation by pro-inflammatory cytokines such as IL-1 and TNF-α [8,9].

IL-6 cytokines have an influence on the proliferation and penetration capacity of squamous tumor cells in vitro [10]. IL-6 is also a regulator of various chronic inflammatory processes that can create better conditions for tumor growth [10]. High levels of cytokine IL-8 in the serum of patients with H&N tumors has shown correlation with the aggressiveness of the tumor and tumor growth [11].

The aim of this pilot study was to find indicators, such as high levels of cytokines, of the severity of mucositis in patients suffering from H&N tumors, who need to receive radiation therapy alone or combined with chemotherapy.

## Patients and Methods

Fifteen patients (12 males, 3 females), median age 51.8 years (range, 18-75 years), with a variety of H&N tumors took part in this pilot study (Table 1). The patients were treated in two hospitals: Hadassah Medical Center in Jerusalem and Rambam Health Care Campus in Haifa. All patients were treated with radiation therapy (60-72 Gy), or radio-chemotherapy. RTOG/EORTC recommendations for post-operative radio-chemotherapy [12,13] were adopted in high-risk patients (Table 2). Chemotherapy was applied as concomitant chemo-radiotherapy, adjuvant chemotherapy or neo-adjuvant chemo-radiotherapy. After approval from the local Institutional Review Board, all patients gave written consent to participate in this study.

**Table 1:** Patient characteristics

Parameter	No. of patients	%
**Site of Disease**		

Nasopharynx	5	33.3

Oropharynx	1	6.7

Hypopharynx	1	6.7

Tongue	1	6.7

Supraglottic larynx	4	26.6

Tonsil	2	13.3

Cervical lymph nodes of unknown primary	1	6.7

**Histology**		

Undifferentiated nasopharyngeal carcinoma	2	13.3

Squamous cell carcinoma	13	86.7

**Stage**		

III	2	13.3

IVa	9	60

IVb	2	13.3

IVc	1	6.7

Loco-regionally advanced, NOS	1	6.7

**Table 2:** Treatment modalities

Parameter	No. of patients	%
**Surgery**		

No	11	73.3

Yes	4	26.7

**Radiotherapy**		

**Total tumor dose, Gy**		

60	2	13.3

66	2	13.3

70	10	66.7

72	1	6.7

**RT Technique**		

2D	3	20

3D	10	66.7

IMRT	2	13.3

**Parotid sparing**		

No	13	86.7

Yes	2	13.3

**RT interruptions**		

Yes (3 d, 6 d, NOS)	3	20

No	12	80

**Chemotherapy**		

**Neoadjuvant chemotherapy**		

No	7	46.6

Yes:		
Cisplatin/5FU^1^	6	40
Carboplatin/5FU^2^	1	6.7
Docetaxel/5FU/Cisplatin^3^	1	6.7

**Concomitant Chemotherapy**		

Carboplatin^4^	1	6.7

Cisplatin^5^	14	93.3

^1 ^Cisplatin 100 mg/m^2^, I.V., day 1; 5-Fluorouracil 1000 mg/m^2 ^I.V., continuous infusion, days 1-5, every 21 days

^2 ^Carboplatin AUC 2, I.V., day 1; 5-Fluorouracil 1000 mg/m^2 ^I.V. continuous infusion, days 1-5, every 21 days

^3 ^Docetaxel 75 mg, Cisplatin 75 mg I.V. day 1; 5-Fluorouracil 750 mg I.V., continuous infusion, days 1-5, every 21 days

^4 ^Carboplatin ACU 2, I.V., weekly

^5 ^Cisplatin 40 mg/m^2 ^I.V., weekly

PEG tube installation was preformed based on clinical evaluation prior, during or at the end of treatment.

All patients had clinical evaluations one week before treatment, in the second and fourth weeks of treatment, and at the end of treatment. WHO common toxicity criteria were used (Grades 1 to 4). Weights were controlled and samples of saliva were collected.

Blood samples were drawn four times (one week before treatment, at the second and fourth weeks of treatment, and at the end of treatment) for the evaluation of inflammatory cytokines IL-1, IL-6, IL-8, TNF-α and the anti-inflammatory cytokine IL-10. This evaluation was done with DPC's ELISA kits in the quantitative "sandwich" enzyme immunoassay technique. A monoclonal antibody specific for the interleukin molecule evaluated was introduced into the wells. Standard antigens together with the serum samples drawn from patients were also introduced into the wells and the interleukin present was bound by the immobilized antibody. After washing away any unbound proteins, the second enzyme-linked antibody specific for the interleukin was added to the wells to "sandwich" the interleukin immobilized during the first incubation. Following a wash to remove any unbound antibody-enzyme reagent, a substrate solution was added to the wells and color developed in proportion to the amount of the cytokine bound in the initial step. By comparing the optical density of the samples to a standard curve, the concentration of the interleukin in unknown samples is determined.

Statistical analysis was done using SPSS (Chicago IL, USA) with Fisher's exact test. The small number of patients forced the use of two-tailed tests to evaluate the statistical hypothesis. Differences between cytokine values at various times were checked with the Mann-Wittney U test. The correlation between cytokine levels and mucositis was checked with the Kruskal Wallis test. Differences between mucositis evaluation for installation of a PEG tube were checked using the Pearson Chi-Square test and Fisher's Exact Test. Statistical difference was defined as p < 0.05.

## Results

Seven (46.7%) of the 15 patients required installation of a PEG tube during radio-chemotherapy, and one female patient needed installation of a PEG tube after ending therapy. Three patients from the group that received PEG tubes needed a break in therapy because of severe side effects. After a short pause, therapy was renewed. One patient died shortly after treatment because of multiple metastases.

The mucositis evaluation showed mucositis grade IV in 30% of the patients after the 4^th ^treatment week. After six weeks of treatment, the number of patients with grade IV mucositis was less (in accordance with boost irradiation to a smaller irradiation field). Patients who showed Grade IV mucositis at week 4 needed to have PEG tube installation (Table 3).

**Table 3:** Mucositis grade correlated to installation of PEG tube

	Need for PEG tube				No need for PEG tube			
Time *	**W0**	**W2**	**W4**	**End**	**W0**	**W2**	**W4**	**End**

**Mucositis grade**								
**1**		50%	0%	0%		66.7%	42.9%	0%
**2**		25%	0%	66.7%		33.3%	28.6%	66.7%
**3**		25%	33.3%	0%		0%	28.6%	33.3%
**4**		0%	66.7%	33.3%		0%	0%	0%

Notes*

W0 = 1 week before treatment

W1 = first treatment week

W2 = second treatment week

W3 = third treatment week

W4 = fourth treatment week

End = end of treatment

Saliva control at rest and after stimulation by patients who needed a PEG tube showed low secretion of saliva during the second and fourth weeks of treatment, compared to before irradiation. Secretions were higher at the end of therapy. In comparison, patients who did not need a PEG tube showed enhanced secretion from the end of the second week (Table 4).

**Table 4:** Saliva secretion at rest and after stimulation in patients with and without PEG tube

	Need for PEG tube				No need for PEG tube			
Time *	**W0**	**W2**	**W4**	**End**	**W0**	**W2**	**W4**	**End**

**Saliva, cc/minute (median)**								
**At rest**	2	0.8	0.3	1	2.1	1	1	1
**After stimulation**	1.7	1	0.3	1	2.5	1	1.3	1.5

Notes*

W0 = 1 week before treatment

W1 = first treatment week

W2 = second treatment week

W3 = third treatment week

W4 = fourth treatment week

End = end of treatment

**Figure 1:**
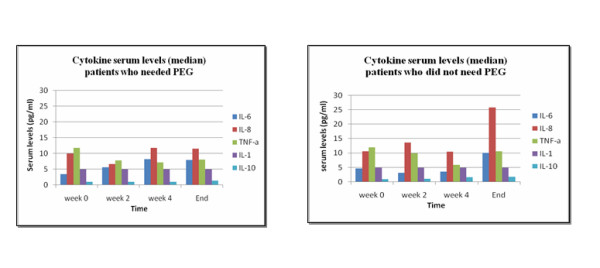
**Cytokine levels during treatment correlated to PEG tube installation**.

The level of cytokines measured before and during therapy showed increased IL-6, decreased TNF-α, and increased IL-8, especially after the second week of therapy. IL-1 and IL-10 did not show any significant changes. A relationship between high levels of IL-6 and the need for a PEG tube was observed. The seven patients who needed a PEG tube had a median IL-6 of 5.6 pg/ml, while patients who did not need a PEG tube had a median of 3.2 pg/ml (p = 0.063). During the second week, a correlation between low levels of IL-8 and PEG tube was found: 6.7 pg/ml with PEG tube versus 13.65 pg/ml without PEG tube (p = 0.031). The other cytokines, IL-10, IL-1, and TNF-α, did not show any correlation with PEG tube installation. The correlation between cytokine levels and PEG tube installation is shown in Figure 1.

**Figure 2:**
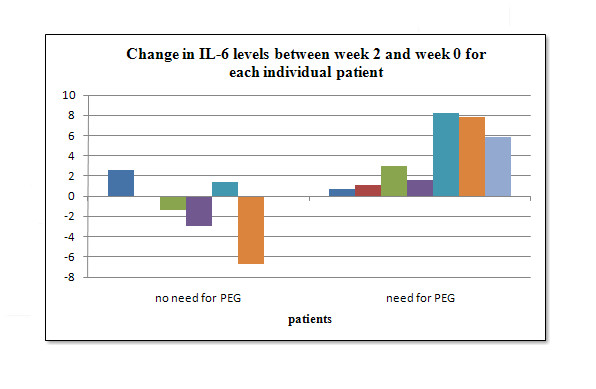
**Change in IL-6 serum levels between second week of treatment and week 0 for each individual patient, correlated to PEG tube installation**.

An even stronger correlation was found between the difference in IL-6 serum levels from baseline values (week 0) to the second week of treatment and the need for PEG tube installation. The patients who needed a PEG tube during treatment had a median difference in IL-6 levels between week 2 and week 0 of 3.0, while patients who did not need a PEG tube had a negative median value of -0.75 (p = 0.022). The correlation between differences in IL-6 levels measured in week 2 as opposed to those measured in week 0 and PEG tube installation is shown in Figure 2.

A relationship between high levels of IL-6 and a high grade of mucositis at week 4 was found (p = 0.081), but no such relationship between IL-1, TNF-α, IL-8 or IL-10 level and mucositis grade was shown.

## Discussion

Radio-chemotherapy leads to various side effects in the irradiated area. These side effects are acute and chronic, and have an influence on the quality of life of the patients [14]. Mucositis is one of the main acute side effects that can lead to therapy delay, compromising optimal treatment. The mucositis usually appears in the second and third weeks of therapy, and the installation of a PEG tube is needed in some cases [4]. Chronic side effects can appear weeks and even years after therapy [15], especially dry mouth, caries, tissue necrosis, fibrosis and radio-osteonecrosis.

We have shown a correlation between inflammatory and anti-inflammatory cytokines and acute mucositis with a need for PEG tube installation. Seven of 15 patients who took part in this pilot study needed PEG tube installation due to the severity of radio-chemotherapy side effects. Weight loss and decreased secretion of saliva was observed in all patients. These two phenomena were enhanced in the seven patients who needed PEG tube installation. The slight enhancement of saliva secretion at the fourth week of treatment can be explained by the small irradiation field ("boost" irradiation). The first phase of mucositis during radio-chemotherapy was characterized by the production of inflammatory cytokines, such as IL-1, TNF-α and IL-6, that coordinate this process in the oral mucosa [2]. It has been demonstrated that the increase of IL-8 and IL-6 and the increase of proteins such as C-reactive protein play an important role in the pathogenesis of H&N squamous cell tumors [8]. Long-term changes in levels of IL-8 and IL-6 after therapy are connected with tumor response to treatment and with tumor progress [16].

This study was aimed at finding a correlation between cytokine levels and the need for PEG tube installation due to mucositis during radiation therapy of H&N squamous cell cancers. We observed high levels of cytokine IL-6 (Figure 1), with a need for PEG tube installation. An even stronger correlation (p = 0.022) between the difference in IL-6 serum levels from baseline values (week 0) to second week of treatment and the need for PEG tube installation was seen (Figure 2). Low levels of IL-8 showed a need for a PEG tube and high levels showed no need (p = 0.031).

We were unable to detect any correlation between IL-1, TNF-α, and IL-10 levels and mucositis. IL-10 is known as an anti-inflammatory cytokine [17], but we did not find any significant changes in IL-10 levels during radio-chemotherapy.

In some reports, elevated levels of TNF-α were documented during irradiation of H&N tumors. This fact leads to suggested treatment with TNF-α cytokines to avoid inflammation [18,19]. In our study, we observed the opposite effect, a decrease of the TNF-α level (Figure 1). TNF-α has an important interaction with other cytokines and hormones. This cytokine influences cell proliferation, various intra-cellular processes and cytostatic effects that, in the presence of IFN-γ, create a cytotoxic effect [6].

We did not observe any change of IL-1 levels in our patients, in contrast to other studies [20] that showed enhancement of IL-1 in patients with mucositis. Fibroblasts, B lymphocytes, and endothelial cells can produce IL-1, a cytokine-induced cytokine which participates in various biological processes, including acute inflammation [20].

During radio-chemotherapy, IL-8 was detected in relatively low levels in patients who needed PEG tube installation. This low level is quite paradoxical, as we presume that high levels of inflammatory cytokines are correlated with severe mucositis. Our study dealt with the reaction of healthy tissues to ionizing irradiation and not in tumors. A similar finding was observed in a study published by Hart et al from Duke University [21] on the influence of irradiation on healthy lung tissue during treatment of bronchial cancer, comparing IL-8 levels prior to irradiation and the probability of developing radiation pneumonitis. All patients showed high levels of IL-8, but a correlation between relatively low levels of IL-8 and an inflammatory process were observed. It was then suggested that IL-8 plays a role against irradiation damage [21].

Our study showed a correlation between high levels of IL-6 and severe mucositis. The presence of this cytokine in blood is known but a level higher than 10 pg/ml is abnormal and can lead to chronic inflammation [22]. IL-6 has an important role in the acute phase response [6] and can be found in several illnesses, such as multiple myeloma and bowel inflammation. IL-6 and its receptor, IL-6R, were found in various tumors, such as in the kidney, lung, ovary, H&N and cervix [9,23]. An *in vitro *study on human squamous cell cancer showed that high concentrations of IL-6 will influence the invasion of tumor cells and that metastasis is possible [24]. The same was demonstrated in an *in vivo *control [9]. Other studies reviewed the correlation between IL-6 before radio-chemotherapy and cancer therapy resistance, including the possibility of metastases. A correlation between IL-6 levels and recurrence was shown, leading to the conclusion that IL-6 can be an indicator of tumor aggressiveness [25].

In a more recent publication, Haddad et al. assessed the use of Amifostine during head and neck chemo-radiation for prevention of mucositis. In their small randomized study, the authors investigated cytokine levels during chemo-radiotherapy. They found an elevation in serum levels of cytokines IL-6, TNF-α and IL-1β which correlated with mucositis severity, and also showed that Amifostine did not reduce mucositis severity. This study confirmed the positive relationship between cytokine levels and mucositis [26].

This pilot study, due to its small sample size, cannot provide a definitive answer to the question of the relationship between levels of IL-6 and IL-8 and severity of mucositis during and after radiotherapy. In addition, it should be emphasized that because of the small sample size, this study may have limitations due to lack of uniformity of radiation dose and technique and the heterogeneity in the chemotherapy regimens employed, especially in the neoadjuvant setting. Therefore, this pilot study can only give an indication, and further studies with larger sample scales are needed, especially to consider the genotype (e.g., SNPs - Single Nucleotide Polymorphism [27]) that can lead to severe mucositis during and after radio-chemotherapy in H&N tumors. A correlation between high IL-6 levels and relatively low IL-8 levels during inflammation, and the severity of radiation-induced mucositis may serve as a prognostic factor to predict the need for PEG tube installation during the first part of treatment, thus placing it prior to the development of complications. Identifying SNPs associated with clinical radio-sensitivity in future studies, in addition to serum cytokine levels, could lead to predicting adverse response to radiotherapy.

## Competing interests

The authors declare that they have no competing interests.

## Authors' contributions

AM participated in the design of the study and clinical evaluations. MK participated in the design of the study and clinical evaluations, and carried out the writing of the manuscript. SB, RAB, AS, TP and MS carried out the clinical evaluations. MSc drafted the manuscript. VB participated in the design of the study and carried out the laboratory analysis. All authors read and approved the final manuscript.
